# Insights from LLIN post-distribution monitoring surveys in the malaria transmission foci of the Dominican Republic: implications for quantification and distribution strategies

**DOI:** 10.1186/s12936-025-05406-6

**Published:** 2025-08-21

**Authors:** Gilda Ventura, María Yinet Santos Félix, Natalia Tejada Bueno, Nicole Michelén Ströfer, Jose Luis Cruz Raposo, Ángel Solís, Rafael German Barrios Parra, Lucía Fernández Montoya

**Affiliations:** 1 Centro de Prevención y Control de Enfermedades Transmitidas por Vectores y Zoonosis (CECOVEZ), Santo Domingo, Dominican Republic; 2 Clinton Health Access Initiative (CHAI), Boston, MA USA

**Keywords:** Malaria elimination, LLINs, Monitoring

## Abstract

**Background:**

Long-lasting insecticidal nets (LLINs) have been distributed and installed in the Dominican Republic since 2008, and they remain the main vector control intervention used to pursue malaria elimination in the country. However, LLIN performance remains unclear due to a lack of monitoring over the past decade.

**Methods:**

A cross-sectional household survey was conducted to monitor LLIN coverage, access, use, physical integrity, washing and drying practices, and the time people go to bed and wake up in the two main malaria foci of the country: Azua (4–6 months post-distribution and installation) and San Juan (one year post-distribution and installation).

**Results:**

The percentage of sleeping spaces that could be covered with a LLIN given the LLINs present in the household was 64% in Azua and 63% in San Juan; with any net, coverage was 75% in Azua and 80% in San Juan. Reported LLIN retention was 88.4% in Azua and 80.9% in San Juan. The percentage of people who had access to sleeping under an LLIN was 58.8% in Azua and 65.4% in San Juan. Among people with LLIN access (people with enough LLINs to cover all sleeping spaces in their household), use was 48.8% in Azua and 75% in San Juan; and overall, LLINs use was 32.3% in Azua and 50.5% in San Juan. Most LLINs remained in serviceable physical condition (Azua: 96.4%, San Juan: 88.9%) but those with holes were not repaired. Most LLINs were washed with aggressive products (Azua: 65%, San Juan: 86%), at a frequency that suggests they will be washed more than twenty times in three years (Azua: 52%, San Juan: 73%), and dried under the sun (Azua: 75%, San Juan: 90%).

**Conclusion:**

Poor washing and drying practices are prevalent in both areas, low LLIN use was observed in Azua and some LLIN coverage gaps were measured in both foci. Urgent behavioural change strategies are needed to improve LLIN care in both foci and to increase LLIN use in Azua, alongside revisions to LLIN quantification methods to ensure full coverage of all sleeping spaces in use during installation. Heterogeneities in LLINs use across foci suggest the need for monitoring use in each distribution area to identify individual gaps and promptly address them.

**Supplementary Information:**

The online version contains supplementary material available at 10.1186/s12936-025-05406-6.

## Background

**Fig. 1:**
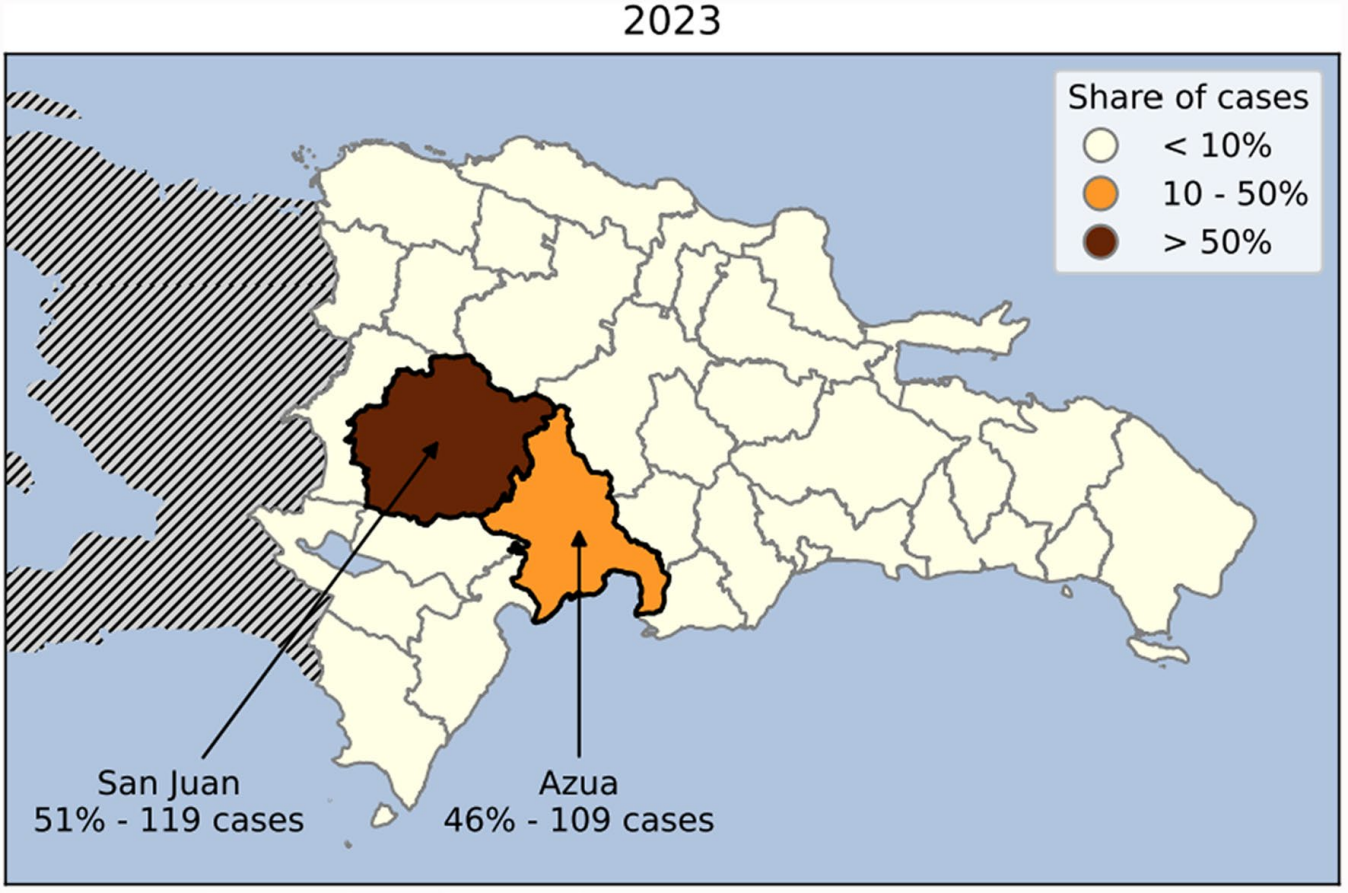
Distribution of indigenous malaria cases in the Dominican Republic in 2023 (data is from Dominican Republic’s national malaria risk stratification)

In the Dominican Republic (DR), malaria has decreased steadily during the past 4 years. There were less than 400 cases reported annually during the last 3 years [1]. As malaria cases decreased in the DR, malaria transmission shifted from urban to rural areas, being currently concentrated in two main rural foci: San Juan and Azua (Fig. 1).

The DR is using pyrethroid-only long-lasting insecticidal nets (LLINs) as their main prevention intervention for malaria. The country has distributed LLINs since 2008 in different foci, with staggered yearly campaigns in 2014–2016, 2019–2023 [2, 3]. However, little is known about the performance of LLINs post-distribution. A case–control study conducted in 2013 in Dajabon (a locality in the northern region of the country that was, in 2013, one of the main active malaria foci) following the first distribution of LLINs in 2008–2009, raised questions about the effectiveness of nets in this context such as whether LLIN use alone was sufficient to provide protection, given that malaria risk appeared similar among LLIN users and non-users; however, the study was not conclusive [4]. A later study conducted in Los Tres Brazos and La Cienaga in 2020 (the two main foci at in that year, which are located in peri-urban areas of the country’s capital, Greater Santo Domingo) showed that, 16 months after LLIN distribution, only 49.4% of people had nets in their home and only 25.1% of those that had nets had sufficient nets for all household members [5].

The protection LLINs confer depends on human behaviour: LLINs provide protection only if people retain and use them. Even when people use them, their protection diminishes as they deteriorate [6–8]. LLIN durability is highly influenced by the users. LLINs’ handling, washing and drying practices can lead to physical damage and reduced bio efficacy [9]. Net retention, use, handling and washing practices vary from country to country and from population to population, as shown through comprehensive studies conducted in Africa and Latin America [9–14]. It is, therefore, important to monitor LLINs post-distribution in different contexts to understand their performance and inform actions that can increase use and prolong the serviceable life of LLINs.

The goal of this programmatic activity was to evaluate LLINs in the main malaria transmission foci several months after distribution, including evaluating LLIN retention, coverage and use. The instructions received by the population during the distribution and installation regarding LLIN use, washing, and drying practices, and the physical integrity of nets were also reviewed. The results of this study help the Ministry of Health (MoH) of the Dominican Republic to improve their LLIN quantification and distribution strategies to maximize LLIN impact.

## Methods

### Study site

The monitoring of vector control interventions was conducted in Azua and San Juan, the two main malaria foci in the Dominican Republic that accounted for 97% of all indigenous malaria cases in 2023 (51% in San Juan and 46% in Azua) (Fig. 1). Azua and San Juan are both rural foci with a high mobile migrant population due to their agricultural nature [15, 16]. Malaria cases are concentrated among agricultural workers. The MoH implements two vector control strategies in both foci: indoor residual spraying (IRS) and the installation of LLINs. The active ingredients used for both interventions are pyrethroids (deltamethrin for IRS and alphacypermethrin for LLINs). In Azua, SafeNet and Interceptor nets were installed in 15 localities between February 9 and May 22, 2023 (between 4 and 6 months prior to this monitoring), and a round of IRS was carried out in 15 localities from April 14 to May 2, 2023. In San Juan, SafeNet nets were installed in 55 localities from August 20 to September 8, 2022 (one year prior to this monitoring) and IRS was conducted in 20 localities from April 18 to May 2, 2023. In both foci, the aim of the LLIN campaign was to install one LLIN per sleeping space in the household, and the aim of the IRS campaign was to spray all sprayable structures. To quantify the needed LLINs based on population estimates, the Dominican Republic assumes that there are five people and three sleeping spaces per household, the country then divides population estimates by five and multiplies them by three to get the number of needed LLINs.

### Survey design

The monitoring of vector control interventions in each focus was carried out by conducting a survey in a random sample of households from localities that received LLINs. The sample size per focus was calculated using the software Epi Info following simple random probability sampling, aiming to obtain a 95% confidence level and a ±6% error in the percentage of houses with enough nets to cover all sleeping spaces and considering that Azua and San Juan had a finite number of households. The total sample size was 361 households in San Juan and 239 households in Azua. The number of houses visited in each locality was calculated based on the proportion of the households that received LLINs in each locality, out of the total number of households in localities that received LLINs. In the absence of an updated population census, the total number of households in each locality was obtained from reports made during the LLIN installation and IRS campaigns. To obtain the interval for selecting households in the field, the total number of households was divided by the number of households to be visited in the locality (e.g. if the locality had 100 households and 10 had to be visited, field teams would interview 1 out of every 10 households they encountered). Fieldworkers started at a random corner of the locality and chose households based on the selection interval (e.g. 1 every 10 households).

Each household received a survey in Spanish (Additional file 1). The survey included questions addressed to the head of the household, questions addressed to a random member of the household, and questions about a randomly selected LLIN in the household. Due to the absence of an updated census of the population, the random member was selected by the surveyor in situ, and so was the random LLIN (considering both installed and uninstalled LLINs present in the household). Several of the questions were verified by the surveyors inside the house by visual inspection of sleeping spaces, nets, and/or net drying places. The physical integrity of the LLINs was verified in situ by extending the LLIN and counting the holes of four different sizes as per WHO guidelines [17]. The surveys were conducted by personnel from the MoH and the Clinton Health Access Initiative (CHAI). The data were collected digitally with the application SurveyCTO installed on the surveyors'phones.

### Data analysis

#### Indicators reported

All indicators shown in Table 1 of Additional file 2 were calculated for each focus and are reported with either their 95% confidence intervals (for proportions) or their standard deviation (for means). As there was a significant number of untreated nets in the two regions, most indicators were reported for both mosquito nets in general (treated and untreated) and for LLINs only. The World Health Organization (WHO) recommended universal coverage indicator is the percentage of households with at least one LLIN for every two people [18, 19]. In this context, the campaigns aimed to distribute one LLIN per sleeping space as the ratio of people per sleeping space is less than two; therefore, the percentage of sleeping spaces that could be protected given the number of LLIN present in the households was reported, assuming that the areas will only achieve full LLIN coverage when all sleeping spaces are covered. Likewise, to report LLIN access, it was not assumed that two people slept under each LLIN, as recommended in the Household Survey Indicators for malaria control [20], but instead the average number of people per sleeping space was calculated and that ratio was used to calculate the LLIN access indicators using otherwise the formula recommended in the Household Survey Indicators for malaria control [20]. To allow for comparison with other monitoring exercises in other countries, the three WHO-recommended indicators were reported in Table 2 of Additional file 2. During the monitoring in San Juan, it was observed that some households had sleeping spaces for visitors, hence in the monitoring conducted in Azua, a question to ask about the number of sleeping spaces used the night before was included. Consequently, the percentage of these spaces that could be protected with the available household LLINs was only reported in Azua, as the question was only added in this focus.

**Table 1:** Estimated indicator values for Azua and San Juan with [95% Confidence interval]

Indicator category	Indicator	Azua (%) (4–6 months post-distribution and installation)	San Juan (%) (1 year post-distribution and installation)
Verification of the quantification method	Average number of people per household	3.98 [2.31–5.65]	3.89 [2.14–5.64]
	Average number of sleeping spaces per household	3.16 [1.96–4.36]	3.15 [2.02–4.28]
	Average number of people per sleeping space	1.4 [0.7–2.1]	1.3 [0.7–1.9]
	Average number of people per sleeping space used the night before	1.5 [0.8–2.1]	#
Retention	Percentage of distributed LLLNs that remain in the households	88.4 [85.5–90.8]	80.9 [78.2–83.3]
Current mosquito net coverage (all nets, treated or not)	Percentage of sleeping spaces that could be covered with a mosquito net given the number of nets in the households	74.5 [71.3–77.4]	80.0 [77.6–82.2]
	Percentage of sleeping spaces used the previous night that could be covered by a mosquito net given the number of nets present in the households	86.7 [83.9–89]	#
	Percentage of sleeping spaces that had a mosquito net visibly installed above them	32.7 [29.5–36.1]	58.7 [55.9–61.5]
Current LLIN coverage	Percentage of sleeping spaces that could be covered with LLIN given the number of LLIN in the households	63.7 [60.2–67.0]	62.5 [59.7–65.3]
	Percentage of sleeping spaces used the previous night that could be covered by a LLIN given the number of LLIN present in the households	74.1 [70.6–77.3]	#
	Percentage of sleeping spaces that had an LLIN visibly installed above them	26.2 [23.6–29.8]	43.6 [40.8–46.5]
LLIN access	Percentage of people that have access to sleeping under a mosquito net (based on the actual number of people per sleeping space)	66.2 [63.1–69.2]	78.1 [75.9–80.2]
	Percentage of people that have access to sleeping under an LLIN (based on the actual number of people per sleeping space)	58.8 [55.6–61.9]	65.4 [62.9–67.9]
Use	Percentage of people who slept under a net the night prior to the survey	40.2 [37.0–43.4]	67.9 [65.5–70.3]
	Percentage of people who slept under a net the night prior to the survey among those living in households with enough nets to cover all sleeping spaces	52.4 [48.1–56.6]	84.2 [81.5–86.5]
	Percentage of people who slept under an LLIN the night prior to the survey	32.3 [29.2–35.3]	50.5 [53.1–47.9]
	Percentage of people who slept under an LLIN the night prior to the survey among those living in households with enough LLIN to cover all sleeping spaces	48.8 [44.3–53.4]	75.0 [71.4–78.4]
Physical integrity	Percentage of LLIN in unserviceable condition (PHI > 642)	3.6 [1.6–7.6]	11.1 [8.0–15.3]
Washing practices	Percentage of LLINs that will be washed more than twenty times in three years if the current washing frequency is sustained	52.4 [44.3–60.3]	72.7 [67.2–77.7]
	Percentage of LLINs washed with aggressive products (e.g. chlorine, shampoo, detergent, bleach)	64.6 [56.5–71.9]	85.8 [81.1–89.5]
Drying practices	Percentage of LLIN dried under the sun	75.5 [68.0–81.8]	90.5 [86.4–93.4]
Instructions received by LLIN recipients	Percentage of households reporting having received washing instructions (any instructions)	52.8 [45.5–60.0]	23.6 [19.2–28.7]
	Percentage of households that received adequate instructions on mosquito net care during distribution and installation (as defined by adequate washing—only water or water and bar soap- and adequate drying instruction—under the shade)	14.9 [8.7–24.1]	8.2 [3.4–17.6]
Human behaviour	Mean time when people went to bed	20:15:58	20:07:37
	Mean time when people woke up	06:56:63	07:18:45

^#^The number of sleeping spaces used the night before was only asked in Azua

Bioefficacy could not be measured because there were no LLINs in-country to replace those that would have been taken to conduct WHO cone bioassays. In lieu, three indicators to gain an understanding of behaviours that could affect bioefficacy were included, namely, the percentage of LLINs that will be washed more than 20 times in 3 years (the WHO requirement for LLINs) [21] if the current frequency of washing is sustained, the percentage of LLINs washed with aggressive products (i.e. bleach, chlorine, detergent or shampoo), and the percentage of LLINs dried under the sun.

### Data analysis

Differences in LLIN use between sexes were evaluated using Chi-square test of inequality. LLIN physical integrity was calculated as per recommended by the WHO [17, 22]. Holes of each of the four following sizes were measured in each LLIN (including all panels and roof):

- Size 1: smaller than a thumb (0.5–2 cm)
- Size 2: larger than a thumb but smaller than a fist (2–10 cm)
- Size 3: larger than a fist but smaller than a head (10–25 cm)
- Size 4: larger than a head (>25 cm)

Then the Proportional Hole Index (pHI) was calculated as:

PHI = (1 × no. of holes of size 1) + (23 × no. of holes of size 2) + (196 × no. of holes of size 3) + (576 × no. of holes of size 4). LLIN were then divided into three categories as shown in Table 1.

## Results

**Fig. 2:**
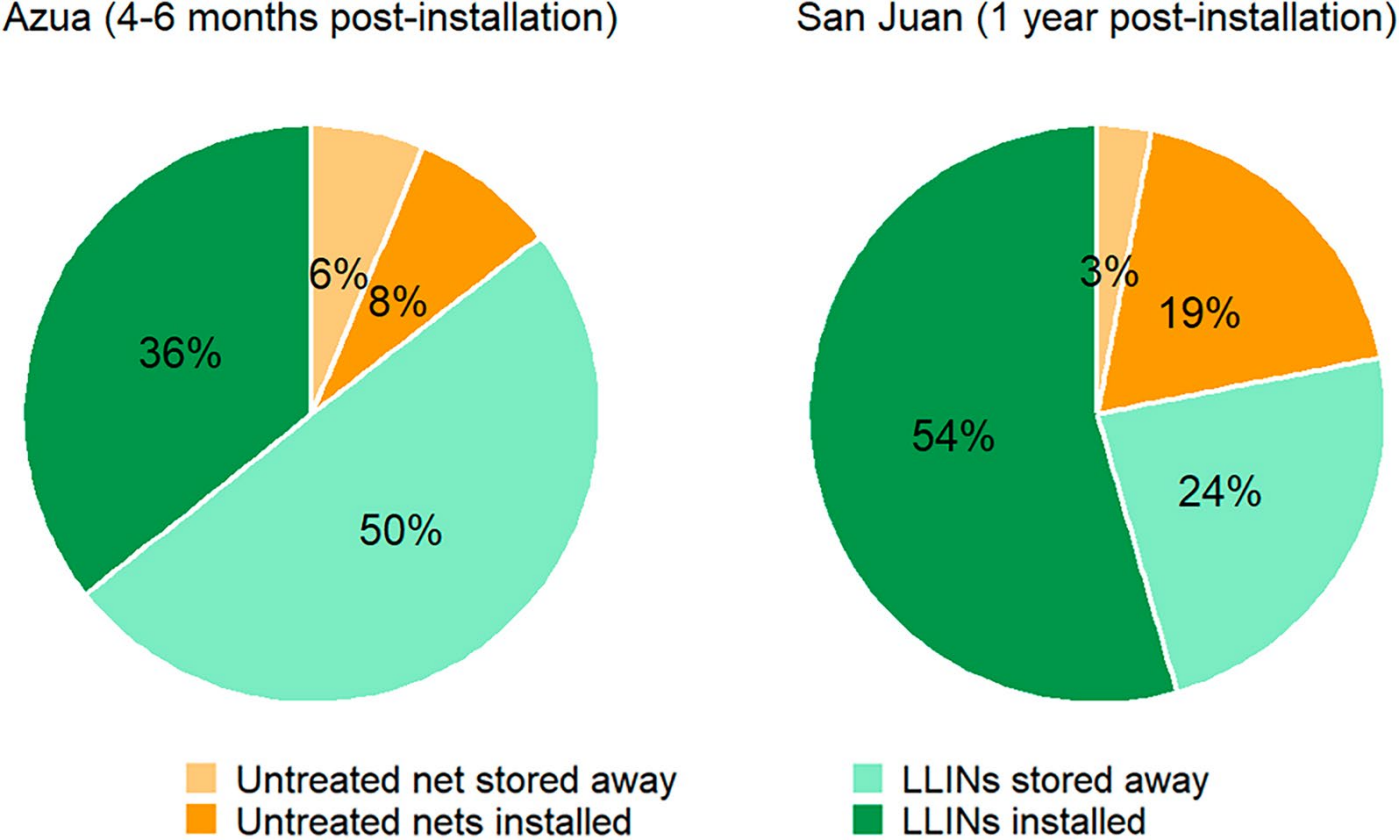
Nets found in the households by treatment status and situation in which they were found

**Fig. 3:**
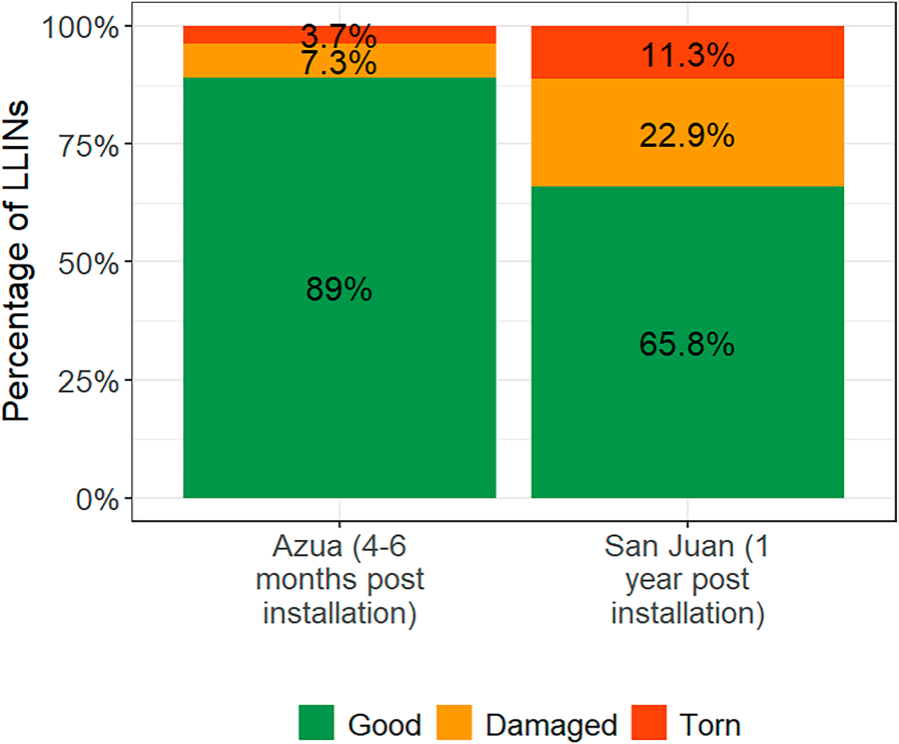
Status of LLINs by physical integrity in Azua and San Juan

Figure 2 presents the percentage of nets found in each status (stored away vs. installed). Table 1 presents the results of all other indicators by region. Both in Azua and San Juan, LLIN use among people with access to an LLIN was higher than use among the overall population and the difference was higher in San Juan than in Azua (San Juan: 50.5 vs. 75%, chi-squared = 102.48, df = 1, *p*-value < 2.2e−16; Azua: 32.3 vs. 48.8%, chi-squared = 35.527, df = 1, *p*-value < 2.516e−9). Bed net use (treated or untreated) was significantly different between men and women in Azua (54.2% for women and 36.7% for men, chi-square = 4.144, df = 1, *p* = 0.04) but not in San Juan (78.9% for women and 75.5% for men, chi-square = 0.046, *p* = 0.8). Figure 3 shows the distribution of LLIN by their physical status. Of the sleeping spaces registered in Azua, 85.9% [83.2–88.2] were used the night before.

## Discussion

This programmatic monitoring of LLINs aimed to evaluate LLIN retention, access, use, physical integrity and washing and drying practices post-distribution, as well as the information received by the population during the campaign regarding net use and care, and population sleeping and wake up times, in the two main malarious regions of the Dominican Republic: Azua and San Juan.

The Dominican Republic aims to cover all sleeping spaces with an LLIN in targeted areas. At the time of the survey, Azua and San Juan were lacking LLINs for 36.3% and 37.5% of their sleeping spaces respectively, and Azua was lacking LLINs for 26% of the sleeping spaces that were used the night before. These are gaps that cannot be justified by attrition (estimated as 11.6% for Azua and 19.1% for San Juan). Suboptimal coverage post-distribution was documented previously in the former two most important foci in the country, La Cienaga and Los Tres Brazos, where (24.6 and 25.6% of people claimed having enough nets for all residents in their home) [5]. The suboptimal coverage could be due to the LLIN quantification method and issues during distribution. To quantify the needed LLINs based on population estimates, the Dominican Republic assumes that there are five people and three sleeping spaces per household, namely the country divides population estimates by five and multiplies them by three to get the number of needed LLINs (population × 0.6). However, in the surveyed foci, the monitoring revealed that there are four people and 3.2 sleeping spaces per household (population × 0.8). This means that the country will need to buy about 33% more LLINs than what they are buying now to adequately cover these two regions in future campaigns. The country could also divide the total population by the average number of people per sleeping space found in this study (1.4 in Azua and 1.3 in San Juan) to get an estimate of the total number of LLINs needed for future campaigns. If LLINs are to be distributed in new areas, the country could conduct a rapid pre-campaign household survey to quantify the needed LLINs. Nevertheless, it is worth questioning the need to cover all sleeping spaces, since the monitoring results showed that 15% of the sleeping spaces in Azua had not been used the night before, so less LLINs may be needed to cover the sleeping spaces effectively in use.

Net access and use were sub-optimal in both foci, but lower in Azua than in San Juan despite the campaign happening only 4–6 months prior to the monitoring survey in Azua (as opposed to a year in San Juan). In Azua, the low use seems to be due to both low access and behavioral factors—such as willingness to use the net— as 66% of the population had access to sleeping under a net (treated or untreated) but only 40% used a net to sleep, and among those living in households with full access, only 52% used a net to sleep. In contrast, in San Juan, the sub-optimal use can be mainly attributed to deficient access, as 78% of people had access to a net and 68% used them, with 84% of those living in households with full access using a net to sleep. Results in San Juan are similar but higher than the results obtained in La Cienaga and Los Tres Brazos in 2020, where most people who had a net for themselves slept under the net the previous night (79.6%)[5]. A high proportion of *bateyes* residents (82.3%) have also been reported to use nets when they have access to them [23]. This suggests that in San Juan, and in other areas in the country, increasing coverage will likely increase net use to an acceptably high level, while in Azua behavioral change campaigns will be needed to increase use. Monitoring data will be needed from each malaria transmission focus receiving LLINs to understand the gaps and identify specific strategies to ensure high use.

Overall, the suboptimal use suggests that the personal protection and community protection currently provided by nets in the country is low compared to what they could provide. This is aggravated by several factors. First, although most of the nets (89%) present in the field are in serviceable condition, 44% already had holes and the population does not repair them (only 15% repaired in Azua and 14% repaired in San Juan) which could presumably lead to further deterioration and reduction in personal protection over time and possibly further reduction in use [24]. Second, most LLINs are being washed with aggressive products (64.6% in Azua and 85.8% in San Juan) and dried under the sun (75.5% in Azua and 90.5% in San Juan), which can cause their bioefficacy to decay rapidly [25]. Unfortunately, bioefficacy could not be measured as the country did not have any left-over LLINs to replace those that would be taken away for bioefficacy assays. In addition to these factors, the recently identified pyrethroid resistance in San Juan, could further hinder the effectiveness of LLINs [26].

Only a small proportion of people (14.9% in Azua and 8.2% in San Juan) recall receiving adequate washing and drying instructions during the campaign, and only 53% in Azua and 24% in San Juan said they had received any instructions on the use or washing of nets during the campaigns. This suggests opportunities to strengthen social and behaviour change communication messaging and training for LLIN distributors on messages to convey during net installation.

Epidemiological data show that both Azua and San Juan have a disproportionately higher number of cases in men than in women [23]. In San Juan, the use of mosquito nets is not significantly different in men and women. By contrast, there is a significant difference in Azua, with women using nets more than men, a factor that could be contributing to the higher burden in the male population.

Although vector behaviour data is not available for Azua or San Juan, data collected in Dajabon (a region 90 km northeast from San Juan, at the border with Haiti) showed that *Anopheles albimanus* is the main vector and is already actively biting by 18:00. Its indoor peak activity time is between 19:00 and 21:00 and its outdoor peak between 20:00 to 22:00, although it continues biting outdoors through the night and indoor until midnight. Since people go to bed at 20:07 in Azua and at 20:15 in San Juan, and if vector behaviour in Dajabon is representative of these two foci, LLINs could prevent a sizable proportion of the bites, but a portion will remain unprevented even if LLIN use increases significantly. The country is implementing IRS in combination with LLINs to complement the protection provided by LLINs, however, the added value of IRS is unclear since the vector resting behaviours observed in Dajabon suggest very little indoor resting and early house exiting [27, 28] and there is no further data collected from Azua or San Juan to evaluate vector resting behaviours there. The added value of IRS should be further evaluated. Additional vector control measures beyond IRS and LLINs are likely needed to complement the protection provided by these two tools.

At regional level, other countries are also facing challenges with LLIN coverage and use at the same time that LLINs have become the main malaria vector control intervention in the region. Nicaragua and Panama are also observing low levels of LLIN use 6 months post-distribution [12]. Similarly, very frequent washing, with harsh products and high rates of drying under the sun, has also been reported in Nicaragua [12] and Guatemala [11]. This shows that, beyond the Dominican Republic, there is an urgent need to regularly monitor LLIN coverage, use and care practices in all countries of Mesoamerica and to promptly address any gaps to ensure adequate performance of LLINs.

The monitoring conducted has three main limitations**.** First, since there was no recent census of the population, the total number of households per locality was estimated using data from the LLIN and IRS campaigns. Hence, the sample size per locality may not be exactly proportional to its population weight, which can affect indicator estimates. Second, there was no record of the number of LLINs that each household received during the campaign, so the attrition indicator could suffer from recall bias, especially in San Juan where LLINs had been distributed a full year prior to monitoring. Finally, the selection of the evaluated LLIN and the interviewed person may have suffered from surveyors’ selection bias affecting the estimation of physical integrity or population sleeping times.

## Conclusions

The survey revealed critical gaps in LLIN coverage, use, LLIN washing and drying practices and in the information provided during the distribution of LLINs both in Azua and San Juan. The monitoring findings highlight the need to implement behavioural change strategies to increase use in Azua, revise LLIN quantification methods to ensure full coverage of all sleeping spaces in use and improve the training of distribution teams to provide adequate information to the populations during the campaign. These gaps should be urgently addressed to optimize the protection provided by LLINs in support of malaria elimination efforts. Finally, the differences in LLIN use observed in both foci underscores the importance of conducting adequate monitoring in all areas receiving LLINs to understand the specific gaps in each area and take appropriate action.

## Supplementary Information

Additional file 1.

Additional file 2.

## Data Availability

The data used to produce this article is available from the repository Figshare with DOI 10.6084/m9.figshare.26010082 This project contains two files: 1) LLIN monitoring data Dominican Republic – 2023- RAW DATA: contains the raw data of the surveys conducted in Azua and San Juan 2) LLIN monitoring data Dominican Republic – 2023- DATA DICTIONARY: is the data dictionary explaining the meaning of each variable in the survey (in Spanish and English). Data are available under the terms of the CC BY 4.0.

## Abbreviations

LLIN: Long-lasting insecticidal net
IRS: Indoor Residual Spraying
CHW: Community health workers
CECOVEZ: Centro de Prevención y Control de Enfermedades Transmitidas por Vectores y Zoonosis (Department for the Prevention and Control of Vector-Borne and Zoonotic diseases of the Ministry of Health of the Dominican Republic)
