# Supplementary material for: Insights from LLIN post-distribution monitoring surveys in the malaria transmission foci of the Dominican Republic: implications for quantification and distribution strategies
Source: Malar J. 2025 Aug 21;24:272. doi: 10.1186/s12936-025-05406-6 (PMC12369129; doi:10.1186/s12936-025-05406-6)
Supplement: Supplementary file 1 — Additional file 1. [file 12936_2025_5406_MOESM1_ESM.docx]

**Additional file 1: Data collection form (Page 1 of 3)**

**FORMULARIO DE MONITOREO DE ACCESO, USO, MANTENIMIENTO E INTEGRIDAD FISICA DE MTILDs**

| **Fecha** | | | **Calle y Numero** | | **Sector** | **Provincia y Foco** | **Responsable** |
| --- | --- | --- | --- | --- | --- | --- | --- |
| **Día** | **Mes** | **Año** |  | |  |  |  |
| **Coordenadas de la vivienda** | | | | **Longitud:** -XX.XXXX | | | **Latitud:** XX.XXXX |
|  |  |  |  |  |  |  |  |

**1. PREGUNTAS AL JEFE O JEFA DE LA VIVIENDA**

| **N° casa** | **Nombre y apellido del o la jefe/a de familia** | **N° de personas que viven regularmente en la vivienda** | | **N° de personas que durmieron en la vivienda la noche anterior** | **N°. de espacios para dormir** | | **N°. de espacios para dormir utilizados la noche anterior** | **N° de mosquiteros en la vivienda** | | **¿Cuántos MTILD recibió en la última campaña de distribución?** |
| --- | --- | --- | --- | --- | --- | --- | --- | --- | --- | --- |
|  |  |  | |  |  | |  |  | |  |
| **Nota:** Si la respuesta a la pregunta sobre el número de mosquiteros en la vivienda es **cero (0)**, pregunte al o la jefe/a de la vivienda  **¿cuál es la razón para que no tenga mosquiteros en la casa?** y escriba la respuesta en el siguiente recuadro: | | | | | | | | | | |
|  | | | | | | | | | | |
| ***Importante:*** *En caso de que no haya mosquiteros en la vivienda y ya sepa la razón, abandone la casa (sin hacer las demás preguntas) y vaya a la siguiente. Empiece nuevamente el cuestionario.* | | | | | | | | | | |
| **Para la siguiente sección del cuestionario, Asegúrese de ver los mosquiteros usted mismo.** | | | | | | | | | | |
| **N° de mosquiteros visiblemente instalados en los espacios para dormir** | | | **De los mosquiteros instalados, ¿cuántos son MTILDs?** | | | **N° de mosquiteros guardados (no instalados)** | | | **De los mosquiteros guardados, ¿cuántos son MTILDs?** | |
|  | | |  | | |  | | |  | |
| **¿Cuáles son las razones para tener los mosquiteros guardados?** | | | | | | | | | | |
|  | | | | | | | | | | |
| **Nota:** Si el número de MTILDs entregados en la campaña anterior, no coincide con los MTILDS que hay en la vivienda, haga las siguientes preguntas al o la jefe/jefa de la vivienda: | | | | | | | | | | |
| **(i) ¿Por qué la cantidad de MTILD entregados es distinta a lo que usted tiene?** | | | | | | | | | | |
|  | | | | | | | | | | |
| **(ii) ¿Le dio algún MTILD a alguien?** | | | | | | | | | | |
|  | | | | | | | | | | |
| **(iii) ¿Cuántos MTILD le dio a otra persona?** | | | | | | | | | | |
|  | | | | | | | | | | |
| **(iv) ¿Alguien le dio MTILD adicionales a usted (luego de la última entrega)?** | | | | | | | | | | |
|  | | | | | | | | | | |
|  |  |  |  |  |  |  |  |  |  |  |

**Additional file 1: Data collection form (Page 2 of 3)**

**FORMULARIO DE MONITOREO DE ACCESO, USO, MANTENIMIENTO E INTEGRIDAD FISICA DE MTILDs**

| **(v) ¿Cuántos MTILD adicionales recibió de otra persona?** | | | | | | | | | | | | | | | | | | | | | | | |  |
| --- | --- | --- | --- | --- | --- | --- | --- | --- | --- | --- | --- | --- | --- | --- | --- | --- | --- | --- | --- | --- | --- | --- | --- | --- |
|  | | | | | | | | | | | | | | | | | | | | | | | |  |
| **(vi) ¿Usted compró MTILD adicionales?** | | | | | | | | | | | | | | | | | | | | | | | |  |
|  | | | | | | | | | | | | | | | | | | | | | | | |  |
| **(vii) ¿Cuántos MTILD adicionales compró?** | | | | | | | | | | | | | | | | | | | | | | | |  |
|  | | | | | | | | | | | | | | | | | | | | | | | |  |
| **(viii) ¿A quién le compró estos MTILD adicionales?** | | | | | | | | | | | | | | | | | | | | | | | |  |
|  | | | | | | | | | | | | | | | | | | | | | | | |  |
| **N° de mosquiteros utilizados la noche anterior** | | | | | **De los mosquiteros utilizados la noche anterior, ¿cuántos son MTILD?** | | | | | | | | **N° de personas que durmieron bajo un mosquitero la noche anterior** | | | | | **De esas personas, ¿cuántas durmieron bajo un MTILD?** | | | | | |  |
|  | | | | |  | | | | | | | |  | | | | |  | | | | | |  |
| **2. PREGUNTAS A UN MIEMBRO DE LA VIVIENDA** | | | | | | | | | | | | | | | | | | | | | | | |  |
| Escoja un integrante de la familia al azar y pregúntele lo siguiente: | | | | | | | | | | | | | | | | | | | | | | | |  |
| **Género (M/F)** | | **Edad** | | **¿Usted durmió bajo un mosquitero anoche?** | | | | | | | **¿Tuvo usted la opción de dormir bajo un mosquitero anoche?** | | | | | **Hora de irse a dormir** | | | | | | **Hora de levantarse** | |  |
|  | |  | | Sí ⧠ | | | | No ⧠ | | | Sí ⧠ | | | No ⧠ | |  | | | | | |  | |  |
| **Nota:** Si la persona no durmió bajo un mosquitero anoche, pero tuvo la opción de hacerlo, pregúntele por la razón y escriba la respuesta: | | | | | | | | | | | | | | | | | | | | | | | |  |
|  | | | | | | | | | | | | | | | | | | | | | | | |  |
| **3. PREGUNTAS SOBRE EL MTILD SELECCIONADO** | | | | | | | | | | | | | | | | | | | | | | | |  |
| Seleccione al azar un MTILD y pregunte lo siguiente sobre el MTILD seleccionado: | | | | | | | | | | | | | | | | | | | | | | | |  |
| **¿Este MTILD fue usado alguna vez para dormir?** | | | **¿Se usó este MTILD para dormir anoche?** | | | | | | **¿El mosquitero se lavó alguna vez?** | | | **¿Con qué frecuencia se lava este MTILD?** | | | **¿Uso algún producto para lavar el mosquitero la última vez que lo lavó?** | | | | | **¿Cuál?** | | | |  |
| Sí ⧠ | No ⧠ | | Sí ⧠ | | | No ⧠ | | | Sí ⧠  No ⧠ | | |  | | | Sí ⧠ | | No ⧠ | | |  | | | |  |
|  |  |  |  |  |  | |  |  | |  |  | |  |  |  |  |  | |  | |  | |  | |

| **¿Dónde puso a secar este MTILD la última vez que lo lavó?** | | **Sol o sombra** | | | **¿Recibió usted instrucciones sobre el lavado y secado de los MTILDs?** | | | | **¿Cuáles?** | | |
| --- | --- | --- | --- | --- | --- | --- | --- | --- | --- | --- | --- |
|  | | Sol ⧠  Sombra ⧠ | | | Sí ⧠ | | | No ⧠ |  | | |
| **Observaciones:**  __________________________________________________________________________________________________________________________________________________________________________________________________________ | | | | | | | | | | | |
| **Additional file 1: Data collection form (Page 3 of 3)**  **FORMULARIO DE MONITOREO DE ACCESO, USO, MANTENIMIENTO E INTEGRIDAD FISICA DE MTILDs** | | | | | | | | | | | |
| **4. Integridad física de MTILDs** | | | | | | | | | | | |
| Usando el mismo MTILD seleccionado, haga las siguientes observaciones: | | | | | | | | | | | |
| **Generalidades del MTILD** | | | | | | **Presencia de agujeros y reparaciones** | | | | | |
| **Marca del MTILD** | | | **Agujeros** | | | **¿Presencia de agujeros reparados?** | | | | **Cantidad de agujeros reparados** | |
|  | | | ⧠ Con agujeros  ⧠ Sin agujeros | | | ⧠ Sí | | ⧠ No | |  | |
| **N° de agujeros en el MTILD** | | | | | | | | | | | |
| **Total tamaño 1**  **(0.5-2cm)** | **Total tamaño 2**  **(2-10cm)** | | | | | | **Total tamaño 3**  **(10-25cm)** | | | | **Total tamaño 4**  **(≥ 25cm)** |
|  |  | | | | | |  | | | |  |
|  | | | | | | | | | | | |
| **Rociado Domiciliario** | | | | | | | | | | | |
| **Su casa fue rociada este año** | | | | **Fecha del último rociado** | | | | | | | |
| ⧠ Si  ⧠ No | | | | Mes_____________________  Año_____________________ | | | | | | | |
|  |  |  |  |  |  |  |  |  |  |  |  |

**Observaciones y sugerencias:** __________________________________________________________________________________________________________________________________________________________________________________________________________________________________________________________________________________________________________________________________________________
