# Supplementary material for: Insights from LLIN post-distribution monitoring surveys in the malaria transmission foci of the Dominican Republic: implications for quantification and distribution strategies
Source: Malar J. 2025 Aug 21;24:272. doi: 10.1186/s12936-025-05406-6 (PMC12369129; doi:10.1186/s12936-025-05406-6)
Supplement: Supplementary file 2 — Additional file 2. [file 12936_2025_5406_MOESM2_ESM.docx]

**Additional file 2:**

**Table 1**: Complete list of indicators calculated during the monitoring of LLINs in Azua and San Juan.

| **Indicator category** | **Indicator** | **Formula** |
| --- | --- | --- |
| **Verification of the quantification method** | Average number of people per household | Average number of residents in the surveyed households |
|  | Average number of sleeping spaces per household | Average number of sleeping spaces in the surveyed households |
|  | Average number of people per sleeping space | Average of the ratio of “Number of residents/Number of sleeping spaces” in surveyed households |
| **Retention** | Percentage of distributed LLINs that remain in the households | Number of the LLINs still present in the surveyed households/Number of LLINs that the surveyed households claimed receiving during the campaign |
| **Situation of the nets in the household** | Percentage of LLINs that were stored away | Number of LLINs that were stored away in the surveyed households/Total Number of LLINs present in the surveyed households |
|  | Percentage of LLINs that were installed above sleeping spaces | Number of LLINs that were installed above sleeping spaces in the surveyed households/Total Number of LLINs present in the surveyed households |
|  | Percentage of untreated nets that were stored away | Number of untreated nets that were stored away in the surveyed households/Total Number of untreated nets present in the surveyed households |
|  | Percentage of untreated nets that were installed above sleeping spaces. | Number of untreated nets that were installed above sleeping spaces in the surveyed households/Total Number of untreated nets present in the surveyed households |
| **Current mosquito net coverage (all nets, treated or not)** | Percentage of sleeping spaces that could be covered with a mosquito net given the number of nets in the households | Number of nets (treated and untreated) that were present in the surveyed households/Number of sleeping spaces in the surveyed households |
|  | Percentage of sleeping spaces used the previous night that could be covered by a mosquito net given the number of nets present in the households | Number of nets (treated and untreated) that were present in the surveyed households/Number of sleeping spaces used the previous night in the surveyed households |
|  | Percentage of sleeping spaces that had a mosquito net visibly installed above them | Number of nets (treated and untreated) installed above sleeping spaces in the surveyed households / Number of sleeping spaces in the surveyed households |
| **Current LLIN coverage** | Percentage of sleeping spaces that could be covered with an LLIN given the number of LLINs in the household | Number of LLINs that were present in the surveyed households/Number of sleeping spaces in the surveyed households |
|  | Percentage of sleeping spaces used the previous night that could be covered by a LLIN given the number of LLIN present in the household | Number of LLINs that were present in the surveyed households/Number of sleeping spaces used the previous night in the surveyed households |
|  | Percentage of sleeping spaces that had an LLIN installed above them | Number of LLINs installed above sleeping spaces in the surveyed households / Number of sleeping spaces in the surveyed households |
| **Net access** | Percentage of people that have access to sleeping under a mosquito net (considering the average number of people that share each sleeping space in the two foci) | Calculation method:   1. Estimate the average number of people per sleeping space in the focus 2. Household by household:   2.1. Multiplied the number of sleeping spaces by the average number of people per sleeping space for the region, to obtain the number of people that could sleep under the household nets  2.2. If the resulting number is larger than the number of residents in the household, take the number of residents in the household as the number that can sleep under the net. If it is smaller, take the resulting number as the number of people that can sleep under a net.  3) Sum the number of people that can sleep under a net across households. Then calculate:  Number of people that can sleep under a net in the surveyed households/Number of residents in the surveyed households |
|  | Percentage of people that have access to sleeping under an LLIN (considering the average number of people that share each sleeping space in the two foci) |  |
| **Net use** | Percentage of people who slept under a net the night prior to the survey | Number of people that slept under a net the night prior to the survey in the surveyed households /Number of people that slept net the night prior to the survey in the surveyed households |
|  | Percentage of people who slept under a net the night prior to the survey among those living in households with enough nets to cover all sleeping spaces | Number of people that slept under a net the night prior to the survey in households with enough nets to cover all sleeping spaces /Number of people that slept net the night prior to the survey in households with enough nets to cover all sleeping spaces |
|  | Percentage of people who slept under an LLIN the night prior to the survey | Number of people that slept under a LLIN the night prior to the survey in the surveyed households /Number of people that slept net the night prior to the survey in the surveyed households |
|  | Percentage of people who slept under an LLIN the night prior to the survey among those living in households with enough LLIN to cover all sleeping spaces | Number of people that slept under a LLIN the night prior to the survey in households with enough nets to cover all sleeping spaces /Number of people that slept net the night prior to the survey in households with enough nets to cover all sleeping spaces |
| **LLIN physical integrity** | Percentage of LLIN that are unserviceable (PhI> 642), damaged (pHI 65-642) and in good condition (pHI ≤64) | Number of LLINs that were unserviceable in the survey households/Number of LLINs evaluated in the surveyed households  Number of LLINs that were damaged in the survey households/Number of LLINs evaluated in the surveyed households  Number of LLINs that were in good condition in the survey households/Number of LLINs evaluated in the surveyed households |
| **LLIN washing practices** | Percentage of LLINs that will be washed more than twenty times in three years if the current washing frequency is sustained | We first estimated the number of washes that LLIN will receive in 20 years based on participant responses (e.g. if washed monthly, it will receive 36 washed in 3 years). Then  Number of LLINs that will be washed more than twenty times in three years/Number of evaluated LLINs |
|  | Percentage of LLINs washed with aggressive products (e.g. chlorine, shampoo, detergent, bleach) | Number of LLINs washed with either chlorine, shampoo, detergent, softener or bleach in the surveyed households/ Number of LLINs evaluated in the surveyed households |
| **LLIN drying practices** | Percentage of LLINs dried under the sun | Number of LLINs dried under the sun in the surveyed households/ Number of LLINs evaluated in the surveyed households |
| **Instructions received by LLIN recipients** | Percentage of households reporting having received any washing instructions during distribution | Number of surveyed households claiming to have received instructions regarding LLIN washing and drying /Number of surveyed households |
|  | Percentage of households that received adequate instructions on mosquito net care during the distribution (as defined by adequate washing - only water or water and bar soap- and adequate drying instruction -under the shade) | Number of surveyed households that received adequate instructions on mosquito net care during the distribution /Number of surveyed households |
| **Human behaviour** | Mean time when people went to bed | Mean time of the day at which respondents claimed going to be the night before the survey |
|  | Mean time when people woke up | Mean time of the day at which respondents claimed waking up on the morning of the survey |

**Table 2:** WHO recommended indicators

| **Indicator** | **Azua (%)**  **(4-6 months post distribution)** | **San Juan (%)**  **(1 year post distribution)** |
| --- | --- | --- |
| Percentage of households with at least one net | 78.2 [72.6-82.9] | 90.1 [86.6-92.7] |
| Percentage of households with at least one LLIN | 69.1 [63.1-74.6] | 77.4 [72.9-81.4] |
| Percentage of households with at least one net for every two people | 65.4 [59.3-71.1] | 70.2 [65.3-74.6] |
| Percentage of households with at least one LLIN for every two people | 51.4 [45.2-57.7] | 43.8 [38.9-48.9] |
| Percentage of people with access to sleeping under a net within their household | 71.7 [68.8-74.5] | 79.4 [77.2-81.4] |
| Percentage of people with access to sleeping under a LLIN within their household | 64.0 [60.8-66.9] | 66.7 [64.2-69.1] |
